# Traditional Chinese medicine-derived monomers protect chondrocytes and delay osteoarthritis progression by regulating mitochondrial quality control

**DOI:** 10.3389/fmolb.2026.1857635

**Published:** 2026-06-08

**Authors:** Xinhui Yang, Ying Li

**Affiliations:** School of Pharmacy, The First Affiliated Hospital of Shihezi University, Shihezi, China

**Keywords:** chondrocytes, mitochondrial biogenesis, mitochondrial quality control, osteoarthritis, traditional Chinese medicine-derived monomers

## Abstract

Osteoarthritis is a common degenerative joint disease characterized by progressive cartilage degeneration, joint structural damage, and functional impairment. Despite its high prevalence, effective therapies capable of slowing structural progression remain limited. Increasing evidence suggests that mitochondrial quality control is a key mechanism in maintaining chondrocyte homeostasis and plays an important role in the development and progression of osteoarthritis. In osteoarthritis chondrocytes, mitochondrial quality control is mainly manifested through impaired mitochondrial biogenesis, dysregulated mitochondrial fusion and fission, abnormal mitophagy, and oxidative stress-related mitochondrial dysfunction. These alterations can lead to reduced ATP production, excessive reactive oxygen species accumulation, decreased mitochondrial membrane potential, enhanced chondrocyte senescence and apoptosis, and extracellular matrix degradation, thereby accelerating osteoarthritis progression. In recent years, traditional Chinese medicine-derived monomers have attracted increasing attention because of their relatively clear chemical composition and multitarget pharmacological activities. Accumulating studies indicate that TCM-derived monomers, such as quercetin, resveratrol, curcumin, baicalin, berberine, and icariin, can protect chondrocytes and delay osteoarthritis progression by regulating key processes involved in mitochondrial quality control. This review focuses on the role of mitochondrial quality control in osteoarthritis chondrocytes and summarizes current research on the protective effects of TCM-derived monomers from the perspectives of mitochondrial biogenesis, mitochondrial dynamics, mitophagy, and oxidative stress-related homeostasis, with the aim of providing a reference for future mechanistic studies and potential therapeutic development.

## 1 Introduction

Osteoarthritis is one of the most common degenerative joint diseases among middle-aged and elderly individuals. It is primarily characterized by joint pain, limited mobility, cartilage degeneration, and decreased joint function, and has become a major cause of reduced quality of life and disability (Katz et al., 2021). With the aging population and the continued rise of risk factors such as obesity, the number of people affected by osteoarthritis and the disease burden continue to increase (GBD 2021 Osteoarthritis Collaborators, 2023). Current clinical management primarily relies on symptomatic measures such as exercise intervention, weight loss, nonsteroidal anti-inflammatory drugs (NSAIDs), and intra-articular injections; however, these approaches have limited efficacy in halting progressive cartilage degeneration and delaying structural disease progression (Courties et al., 2024). Consequently, identifying new intervention targets by focusing on key pathological mechanisms of osteoarthritis has become a major direction for basic and translational research in this field (Cheung et al., 2024).

Chondrocytes are the sole cellular component of articular cartilage, and disruptions in their homeostasis are considered a central mechanism in the onset and progression of osteoarthritis (Cheung et al., 2024; Cai et al., 2025). Recent studies have shown that mitochondria not only contribute to the energy supply of chondrocytes but also directly influence redox status, inflammatory responses, matrix synthesis and degradation, and cell survival (Adam et al., 2024). In the osteoarthritis microenvironment, factors such as inflammatory cytokines, mechanical stress, aging, and metabolic abnormalities can collectively lead to impaired mitochondrial function, manifested as reduced ATP production, reactive oxygen species (ROS) accumulation, decreased membrane potential, and mitochondrial DNA damage (Qi et al., 2024). These changes further promote chondrocyte senescence, apoptosis, and enhanced catabolism, thereby exacerbating cartilage matrix degradation and joint degeneration (Diekman and Loeser, 2024).

Mitochondrial quality control is a key mechanism for maintaining the stability of mitochondrial numbers and function, primarily encompassing processes such as mitochondrial biogenesis, mitochondrial dynamics, mitochondrial autophagy, and the maintenance of oxidative stress homeostasis (Wang et al., 2023). When these processes are disrupted, damaged mitochondria cannot be repaired or cleared in a timely manner, leading to persistent oxidative damage and metabolic imbalance, which in turn drives the progression of osteoarthritis (Bonanni et al., 2025). Therefore, understanding the mechanisms of chondrocyte damage from the perspective of mitochondrial quality control contributes to a more systematic understanding of the pathological basis of osteoarthritis.

**FIGURE 1:**
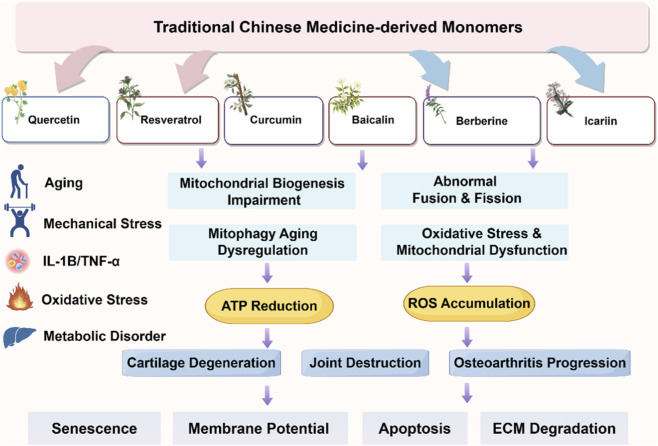
Traditional Chinese medicine-derived monomers protect chondrocytes and delay osteoarthritis progression by regulating mitochondrial quality control. Factors such as aging, mechanical stress, inflammatory stimulation, oxidative stress, and metabolic disorders can induce impaired mitochondrial biogenesis, an imbalance in mitochondrial fusion and fission, abnormal mitochondrial autophagy, and oxidative stress-related mitochondrial dysfunction. This further leads to reduced ATP production, ROS accumulation, decreased membrane potential, increased cellular senescence and apoptosis, and extracellular matrix degradation, ultimately promoting cartilage degeneration, joint destruction, and the progression of osteoarthritis. TCM-derived monomers, such as quercetin, resveratrol, curcumin, baicalin, berberine, and icariin, can exert chondroprotective effects by maintaining mitochondrial homeostasis through intervention in these key pathways.

TCM-derived monomers are characterized by relatively well-defined compositions, multi-target pharmacological actions, and ease of mechanism-based research; consequently, studies on their application in the prevention and treatment of osteoarthritis have increased in recent years (Lei and Zhou, 2025). Existing research suggests that certain TCM-derived monomers can mitigate chondrocyte damage induced by inflammatory stimuli and delay the progression of osteoarthritis by regulating mitochondrial autophagy, alleviating oxidative stress, and improving mitochondrial dysfunction (He and He, 2023). Additionally, animal studies have shown that TCM-derived monomers may also improve joint structural damage and pain symptoms through metabolism-related pathways such as AMPK, suggesting they possess potential for disease intervention (Li et al., 2022). Based on this, systematically reviewing the research progress on how TCM-derived monomers protect chondrocytes and delay the progression of osteoarthritis by regulating mitochondrial quality control is of practical significance for summarizing existing evidence, clarifying the primary mechanisms of action, and providing insights for future research (Figure 1).

## 2 TCM-derived monomers regulate mitochondrial biogenesis to protect chondrocytes

Within the mitochondrial quality control system, mitochondrial biogenesis is a fundamental process for maintaining mitochondrial numbers, renewing damaged mitochondria, and ensuring stable energy metabolism. Its core regulatory axis primarily includes molecules such as AMPK, SIRT1, PGC-1α, NRF1, NRF2, and TFAM (Blanco and Fernández-Moreno, 2020). Among these, PGC-1α is considered a key node linking energy sensing to mitochondrial transcriptional regulation. It promotes mitochondrial DNA replication, respiratory chain assembly, and the maintenance of oxidative phosphorylation function by activating downstream nuclear respiratory factors and mitochondrial transcription-related proteins (Wang et al., 2023; Alvarez-Garcia et al., 2017). In normal chondrocytes, this process helps maintain ATP production, membrane potential stability, and the cell’s ability to adapt to mechanical loads and inflammatory stimuli (Zhou et al., 2022). However, in osteoarthritis, chondrocytes are chronically exposed to an environment characterized by inflammatory factors, oxidative stress, aging, and metabolic abnormalities. Consequently, mitochondrial biogenesis capacity often declines, manifested by downregulated expression of molecules such as PGC-1α, TFAM, and NRF1, with concurrent impairment of mitochondrial number and function (Qi et al., 2024; Wu et al., 2024). Early studies have suggested that insufficient mitochondrial biogenesis in human osteoarthritic chondrocytes is closely associated with enhanced catabolism, and that this alteration is not merely a concomitant phenomenon but may directly contribute to the formation of the pathological phenotype of chondrocytes (Wang et al., 2015). Subsequent studies further demonstrated that AGEs, chronic low-grade inflammation, and energy stress can inhibit AMPKα and SIRT1 activity, thereby impairing PGC-1α-mediated compensatory mitochondrial renewal. This leads to increased susceptibility of chondrocytes to ROS accumulation, mitochondrial DNA damage, and reduced matrix synthesis capacity (Oshitari, 2023). These findings suggest that impaired mitochondrial biogenesis is not a marginal change in osteoarthritis, but rather a critical pathological mechanism closely associated with the disruption of chondrocyte homeostasis (Chen L. et al., 2025).

From a mechanistic perspective, mitochondrial biogenesis defects further exacerbate the metabolic vulnerability of chondrocytes in osteoarthritis. On the one hand, insufficient mitochondrial turnover leads to impaired respiratory chain complex function and reduced ATP synthesis, thereby affecting the basic energy supply required for chondrocytes to maintain matrix synthesis and ion homeostasis (Fu et al., 2024). On the other hand, the persistent accumulation of aged or damaged mitochondria enhances electron transport chain leakage and ROS production, leading to further damage to lipids, proteins, and mitochondrial DNA, thereby creating a vicious cycle (Wu et al., 2026). In this process, the inhibition of the AMPK/SIRT1/PGC-1α axis is particularly significant, as this pathway influences mitochondrial biogenesis and is closely associated with antioxidant defense, anti-inflammatory effects, and cell survival (Chen J. et al., 2025). Previous reviews have indicated that decreased PGC-1α levels not only impair mitochondrial renewal capacity but also indirectly promote the upregulation of MMPs and the loss of type II collagen and aggrecan, thereby driving cartilage degeneration (Li et al., 2026). Furthermore, SIRT1, as a key deacetylase regulator of PGC-1α, is often downregulated or hypoactive in osteoarthritis, and its downregulation is associated with mitochondrial metabolic dysfunction, cellular senescence, and amplified inflammation (Liao et al., 2024). Therefore, targeting the restoration of mitochondrial biogenesis can not only improve energy metabolism but may also, to some extent, alleviate inflammatory and catabolic responses in chondrocytes (Tan et al., 2025) (Figure 1).

In recent years, research on TCM-derived monomers in this area has gradually increased. A growing body of evidence suggests that these compounds can improve insufficient mitochondrial biogenesis in chondrocytes by activating AMPK, enhancing SIRT1 activity, and upregulating PGC-1α and its downstream target molecules (Lei and Zhou, 2025; Dzhalilova and Makarova, 2022). Quercetin is a representative compound frequently cited in this field. Existing animal and cellular studies have shown that quercetin can upregulate the AMPK/SIRT1 signaling pathway, thereby improving impaired mitochondrial function, reduced membrane potential, and elevated ROS levels in osteoarthritis models, while simultaneously promoting the restoration of mitochondrial biogenesis-related protein expression (Wang J. et al., 2025). This study also suggests that chrysanthemum intervention reduces pathological damage in cartilage tissue, indicating that its effects are not limited to *in vitro* molecular changes but are consistent with protective effects at the tissue level (Ma et al., 2021). In addition to chrysanthemum, resveratrol, as a representative polyphenol, is also widely recognized as being closely associated with the regulation of SIRT1 and PGC-1α (Li et al., 2021). Although some early studies reported conflicting findings regarding its effects on the differentiation status of chondrocytes, recent mechanistic reviews generally continue to regard resveratrol as one of the key natural compounds that improves mitochondrial function, alleviates oxidative stress, and helps maintain chondrocyte homeostasis (Fan et al., 2024).

In addition to the aforementioned components, TCM-derived monomers, such as curcumin, icariin, baicalin, and berberine, have also been reported to improve pathological changes associated with mitochondrial biogenesis in various ways (Hridayanka et al., 2024). Curcumin exhibits clear anti-inflammatory and mitochondria-protective effects in osteoarthritis models; recent studies suggest it can improve mitochondrial homeostasis, alleviate oxidative stress, and reduce the extent of chondrocyte damage (Wang et al., 2024). Research on icariin has focused more on its autophagy-inducing and anti-ferroptotic effects; however, its upstream signaling involves the PI3K/Akt/mTOR pathway, energy metabolism, and cellular stress networks, which overlap with the regulation of mitochondrial homeostasis, suggesting that it may indirectly influence mitochondrial renewal and functional maintenance (Chen Y. et al., 2022). Scutellarin has been reported to protect chondrocytes against IL-1β-induced damage by improving mitophagy and metabolic status. These findings also suggest that mitochondrial biogenesis and mitochondrial clearance processes are not mutually exclusive in interventions involving TCM monomers (He and He, 2023; You et al., 2025). Overall, existing studies indicate that the regulation of mitochondrial biogenesis in osteoarthritis chondrocytes by TCM-derived monomers primarily focuses on the AMPK/SIRT1/PGC-1α axis, further influencing TFAM, respiratory chain function, ROS levels, and cellular energy status. However, the models, dosages, and observational indicators used in different studies are not entirely consistent, and systematic validation studies directly focused on mitochondrial biogenesis remain limited. This suggests that future research needs to be further strengthened in terms of mechanistic depth and the completeness of evidence.

Overall, mitochondrial biogenesis and mitochondrial fusion-fission balance are closely interconnected processes within the mitochondrial quality control network, and together they help maintain mitochondrial function and chondrocyte homeostasis in osteoarthritis.

## 3 TCM-derived monomers regulate mitochondrial dynamics to protect chondrocytes

Mitochondrial dynamics primarily refer to the process by which mitochondria maintain a dynamic equilibrium between fusion and fission; this process plays a fundamental role in chondrocyte energy metabolism, redox homeostasis, and stress adaptation (Tan et al., 2025; Shen et al., 2024). During mitochondrial fusion, outer membrane fusion proteins MFN1 and MFN2, together with the inner membrane fusion protein OPA1, jointly maintain the integrity of the mitochondrial network, enabling mitochondria to exchange contents, dilute localized damage, and maintain the efficiency of oxidative phosphorylation (Ashrafi and Schwarz, 2013). During mitochondrial fission, DRP1 is recruited to the outer mitochondrial membrane to mediate mitochondrial fragmentation; this process is not inherently harmful but rather an essential component of mitochondrial renewal and cellular stress adaptation (Ansari et al., 2022). However, in osteoarthritis, this balance often shifts from moderate regulation to persistent imbalance, manifesting as abnormal DRP1 activation, excessive mitochondrial fragmentation, and reduced fusion capacity (Shao et al., 2024). Previous studies have shown that elevated levels of p-DRP1 in osteoarthritic chondrocytes are closely associated with decreased mitochondrial membrane potential, ROS accumulation, and enhanced apoptosis, suggesting that abnormal fission is a key driver of mitochondrial damage in chondrocytes (Jin et al., 2018). Concurrently, decreased expression of MFN2 and OPA1 compromises mitochondrial network stability, making damaged mitochondria more prone to accumulation and amplifying inflammatory and catabolic responses (Xu et al., 2020). These changes are not merely accompanying phenomena at the ultrastructural level but are closely associated with chondrocyte phenotypic drift, matrix homeostasis imbalance, and the progression of osteoarthritis (Wan et al., 2021).

From a pathophysiological perspective, mitochondrial dysregulation can exacerbate cartilage degeneration through multiple mechanisms (Deng et al., 2025). On the one hand, excessive fragmentation leads to disruption of the mitochondrial network and reduced respiratory chain efficiency, resulting in decreased ATP production and impairing the basic energy supply required for chondrocytes to maintain matrix synthesis (Sun Z. et al., 2024). On the other hand, fragmented mitochondria are more prone to electron leakage and excessive ROS production, which further induces lipid peroxidation, mitochondrial DNA damage, and cytochrome c release, thereby driving the activation of the mitochondrial-dependent apoptosis pathway (Lin Z. et al., 2024). ERK1/2-mediated activation of DRP1 has been shown to directly promote mitochondrial fragmentation and apoptosis in chondrocytes stimulated by IL-1β; this finding provides relatively direct evidence for a causal link between abnormal fragmentation and cellular damage in osteoarthritis (Ansari et al., 2022; Cao and Zheng, 2019). Furthermore, a study on aging chondrocytes and osteoarthritis models demonstrated that abnormal changes in MFN2 are associated with metabolic dysregulation and enhanced inflammatory responses, suggesting that fusion defects also contribute to the maintenance of pathological states in chondrocytes (Chang et al., 2020). Recent reviews generally agree that mitochondrial dysregulation in osteoarthritis is not an isolated event but is intertwined with oxidative stress, insufficient mitochondrial autophagy, cellular senescence, and amplified inflammation, which collectively drive the functional decline of chondrocytes (Jiang et al., 2026). Therefore, restoring the balance between fusion and fission—particularly by inhibiting abnormal DRP1 activation and improving MFN2- and OPA1-mediated fusion capacity—has become a key direction for mitochondrial-targeted interventions (Wu et al., 2025).

**FIGURE 2:**
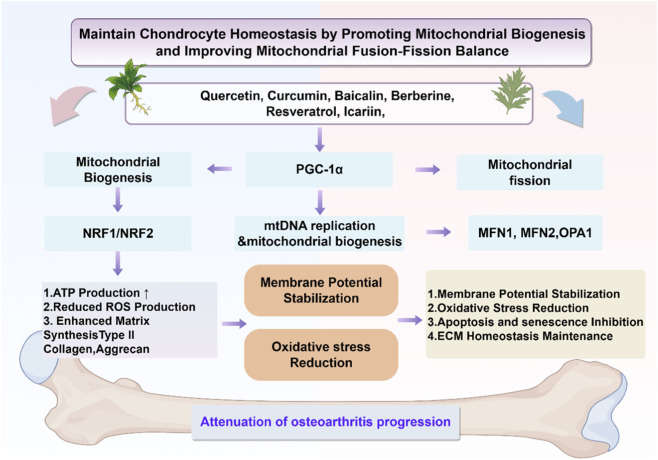
Traditional Chinese medicine-derived monomers maintain chondrocyte homeostasis by promoting mitochondrial biogenesis and improving mitochondrial fusion-fission balance. TCM-derived monomers may promote mitochondrial biogenesis by regulating PGC-1α and its related signaling pathways, thereby enhancing mtDNA replication, mitochondrial renewal, and the expression of downstream molecules such as NRF1, NRF2, and TFAM. In parallel, these compounds may help restore mitochondrial fusion-fission balance by modulating key dynamic regulators, including MFN1, MFN2, OPA1, and DRP1. Through these effects, TCM-derived monomers contribute to improved ATP production, reduced ROS accumulation, stabilization of mitochondrial membrane potential, and preservation of extracellular matrix homeostasis, thereby delaying osteoarthritis progression.

In recent years, TCM-derived monomers have demonstrated a certain research foundation in regulating this pathway, but the evidence remains largely preliminary (Bhogal et al., 2018) (Figure 2).

Quercetin is a representative TCM-derived monomer that has been shown to reduce ROS accumulation, improve mitochondrial dysfunction, and alleviate chondrocyte damage in osteoarthritis (Lei and Zhou, 2025; Pan et al., 2021). Although this study focused more on AMPK/SIRT1-mediated mitochondrial function recovery, given the close coupling between dynamic equilibrium and biogenesis as well as oxidative stress, the results still suggest that quercetin may indirectly correct abnormal fission states by holistically improving mitochondrial homeostasis (Wang R. et al., 2025). Curcumin has relatively more robust evidence in this regard. Previous studies have shown that curcumin can reduce oxidative stress, apoptosis, and cartilage damage in osteoarthritis models, and helps maintain the expression of proteins related to mitochondrial function (Jin et al., 2022). A 2024 study further suggests that curcumin can improve mitochondrial dysfunction and reduce chondrocyte damage through p38 MAPK-related mechanisms, indicating that its effects are not limited to simple anti-inflammatory action but are related to the maintenance of mitochondrial homeostasis (Wang et al., 2024; Peng et al., 2021). Additionally, a 2025 animal and cellular study found that following combined curcumin and omega-3 intervention, pain and cartilage damage were reduced in a MIA-induced osteoarthritis model, while mitochondrial-related markers such as COX IV and TOMM20 increased, suggesting an overall beneficial effect on mitochondrial status (Jhun et al., 2025). Although these studies did not all directly measure DRP1, MFN2, or OPA1, the results suggest that curcumin-based interventions provide some support for inhibiting the accumulation of mitochondrial damage and stabilizing the mitochondrial network (Yan et al., 2019).

In addition to quercetin and curcumin, TCM-derived monomers, such as baicalin and icariin, may also exert protective effects by influencing processes related to mitochondrial dynamics (Hridayanka et al., 2024; Lu et al., 2025). Baicalin has been shown in osteoarthritis studies to reduce cartilage damage and cell death; existing evidence focuses primarily on its anti-ferroptotic and antioxidant effects, although these actions are closely linked to the maintenance of mitochondrial structural integrity (Wan et al., 2023). Early cellular studies also suggest that baicalin can improve mitochondrial dysregulation and alleviate oxidative stress and apoptosis. Although this study was not conducted in an osteoarthritis model, its results provide a basis for understanding how baicalin-derived monomers regulate the balance between fusion and fission (Wan et al., 2023; Si and Lai, 2024). In osteoarthritis, icariin primarily acts by inhibiting chondrocyte apoptosis, regulating autophagy, and alleviating cartilage degeneration (Tan et al., 2021). Recent studies have also suggested that icariin can inhibit chondrocyte ferroptosis and improve articular cartilage damage, indicating that it exerts a certain buffering effect on cell stress associated with mitochondrial damage (Xiao et al., 2024). Overall, research on mitochondrial dynamics involving TCM-derived monomers still faces a notable limitation: while most studies emphasize improving mitochondrial function, reducing ROS, and inhibiting apoptosis, there remains a scarcity of research directly focusing on DRP1 phosphorylation, changes in MFN2/OPA1 expression, and quantitative analysis of mitochondrial morphology (Zhang SO. et al., 2026). This implies that current evidence primarily supports the ability of TCM-derived monomers to mitigate pathological consequences associated with mitochondrial dysregulation; however, more systematic mechanistic validation is required to determine whether they directly restore the balance between fusion and fission (Li et al., 2026; Lin S. et al., 2024). However, based on current research trends, mitochondrial dynamics are increasingly recognized as a critical link connecting inflammatory stimuli, metabolic disorders, cell death, and cartilage degeneration; the therapeutic value of TCM-derived monomers in this context warrants further in-depth investigation and study (Chen S. et al., 2022) (Figure 2).

## 4 TCM-derived monomers regulate mitophagy to protect chondrocytes

Within the mitochondrial quality control system, mitochondrial autophagy is a critical process for selectively identifying and eliminating damaged mitochondria, playing a key role in maintaining the mitochondrial number, energy metabolism, and redox homeostasis of chondrocytes (Lin J. et al., 2024). Recent studies generally agree that osteoarthritis is not merely a simple process of cartilage wear but a complex pathological state accompanied by mitochondrial dysfunction, accumulated oxidative stress, and disrupted mitochondrial quality control, with mitochondrial autophagy imbalance being a particularly important component (Ma et al., 2024). Under normal conditions, moderate mitochondrial autophagy helps promptly clear depolarized or damaged mitochondria, limiting the continuous accumulation of ROS, preventing mitochondrial DNA damage, and avoiding the amplification of pro-apoptotic signals, thereby maintaining the basic survival state of chondrocytes and the metabolic balance of the extracellular matrix (Collins et al., 2018). However, in the osteoarthritis microenvironment, inflammatory factors, abnormal mechanical loading, aging, and metabolic stress can collectively disrupt the mitochondrial autophagy pathway, making it difficult to promptly replace damaged mitochondria, and ultimately driving chondrocyte functional decline and exacerbating articular cartilage degeneration.

From a molecular mechanism perspective, the most frequently discussed mitochondrial autophagy pathways in osteoarthritis primarily include the PINK1/Parkin-dependent pathway and receptor-mediated pathways involving BNIP3, FUNDC1, and others (Kim et al., 2021). In this process, PINK1 accumulates at the outer membrane following a decline in mitochondrial membrane potential and recruits Parkin to mediate the ubiquitination of relevant substrates, thereby promoting the recognition and engulfment of damaged mitochondria by autophagosomes. This process is considered one of the core mechanisms maintaining mitochondrial homeostasis in chondrocytes (Zhuang et al., 2024). FUNDC1-related mitochondrial autophagy is more involved in the regulation of mitochondrial renewal under cellular stress conditions; its dysfunction also leads to the accumulation of damaged mitochondria and metabolic disorders within chondrocytes (Fang et al., 2023). Recent studies have also shown that certain non-coding RNAs and stress-related proteins can promote the progression of osteoarthritis by inhibiting mitochondrial autophagy; for example, upregulation of mt-tRF3b-LeuTAA and PARP12 has been shown to be associated with suppressed mitochondrial autophagy, mitochondrial dysfunction, and exacerbated cartilage degeneration (Long et al., 2025; Deng et al., 2024). These findings suggest that insufficient mitochondrial autophagy is not a single molecular change but rather a critical amplifying step in the chondrocyte damage network of osteoarthritis.

However, when it comes to osteoarthritis, more mitochondrial autophagy is not necessarily better. Current research suggests that moderate mitochondrial autophagy has a protective effect, whereas persistent deficiency leads to the accumulation of damaged mitochondria, and excessive activation may result in excessive mitochondrial loss and energy supply deficiencies. Therefore, the key to this process lies not in simply enhancing autophagy, but in restoring a level of mitochondrial turnover that matches the metabolic demands of chondrocytes. This point is particularly important for understanding the mechanisms of action of single compounds derived from traditional Chinese medicine, as most natural bioactive components do not simply elevate a single autophagy molecule but rather act through upstream stress pathways such as AMPK, SIRT3, and PI3K/Akt/mTOR to restore a relative balance among mitochondrial autophagy, oxidative stress, and cell survival (Zhang et al., 2022).

Among single compounds derived from traditional Chinese medicine, curcumin is one of the best-studied examples to date. Existing studies have shown that curcumin promotes mitochondrial autophagy by activating the AMPK/PINK1/Parkin pathway, thereby improving mitochondrial damage, ROS accumulation, and apoptosis in osteoarthritis chondrocytes, and reducing cartilage structural damage in animal models (Toegel et al., 2008). These findings suggest that the protective effects of curcumin are not limited to anti-inflammatory mechanisms but are also directly related to enhanced clearance of damaged mitochondria. The evidence for baicalin in this regard is also quite clear. Studies have shown that baicalin can inhibit the PI3K/Akt/mTOR signaling pathway in an IL-1β-induced osteoarthritis cell model, activate PINK1/Parkin-related mitochondrial autophagy, and alleviate inflammatory responses, oxidative damage, and chondrocyte apoptosis (Chen et al., 2018). Compared to the previous sections on biogenesis and kinetics, this category of studies more directly links TCM-derived monomers to the core pathways of mitochondrial autophagy, thereby offering a more targeted mechanistic understanding.

In addition to curcumin and baicalin, several other single compounds derived from traditional Chinese medicine have been reported in recent years to exert chondroprotective effects through similar mechanisms. Coptisine activates PINK1/Parkin-mediated mitochondrial autophagy, inhibits IL-1β-induced chondrocyte inflammation, and reduces articular cartilage damage in a rat osteoarthritis model (75). Protocatechuic aldehyde has been shown to modulate PINK1/Parkin-mediated mitochondrial autophagy, alleviate the senescent phenotype in chondrocytes, and delay cartilage degeneration (Jie et al., 2024). Acetylgingerone, on the other hand, can reduce pyroptosis and improve the progression of osteoarthritis by promoting PINK1/Parkin signaling, suggesting that mitochondrial autophagy may also be cross-regulated with inflammatory cell death (Zhang et al., 2025). A 2026 study further suggested that plant-derived glycosides can promote mitochondrial autophagy by activating the Sirt3/PINK1/Parkin axis, thereby alleviating chondrocyte inflammation and joint degeneration (Zhang H. et al., 2026). Overall, existing studies have consistently shown that the primary mechanisms by which TCM-derived monomers regulate mitochondrial autophagy involve promoting the clearance of damaged mitochondria, reducing ROS burden, and improving membrane potential and mitochondrial function, thereby inhibiting inflammation, aging, apoptosis, and matrix degradation (Sun Y. et al., 2024). However, experimental models, intervention doses, and evaluation criteria remain inconsistent across different studies. Some studies still rely primarily on changes in the expression of a few proteins to assess mitochondrial autophagy, lacking systematic validation of autophagy flux and mitochondrial morphology. This indicates that further refinement is needed in this area.

## 5 TCM-derived monomers alleviate oxidative stress and maintain mitochondrial homeostasis in chondrocytes

**FIGURE 3:**
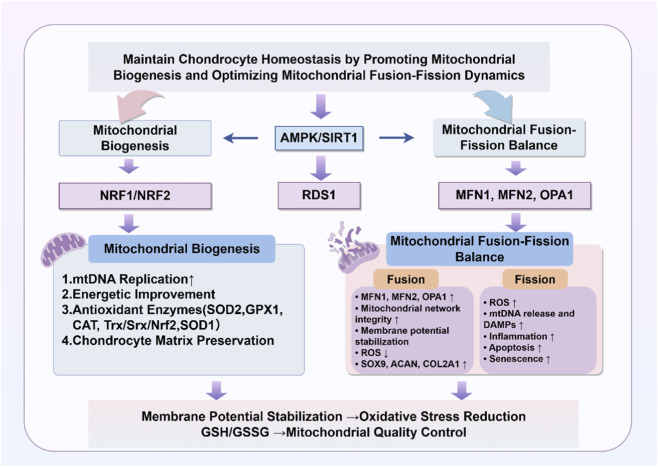
Traditional Chinese medicine-derived monomers maintain chondrocyte homeostasis via AMPK/SIRT1-mediated mitochondrial biogenesis and fusion-fission dynamics. TCM-derived monomers regulate mitochondrial quality control in osteoarthritis chondrocytes through the AMPK/SIRT1 signaling axis. Activation of this pathway promotes mitochondrial biogenesis through downstream regulators such as PGC-1α, NRF1, NRF2, and TFAM, while also contributing to the maintenance of mitochondrial dynamics and functional homeostasis. In addition, AMPK/SIRT1-related regulation may enhance antioxidant defense systems, improve mitochondrial membrane potential and energy metabolism, reduce ROS accumulation, and suppress apoptosis, senescence, and inflammatory responses. Through these coordinated effects, TCM-derived monomers help preserve chondrocyte homeostasis and protect cartilage from degeneration.

Oxidative stress is a major driver of mitochondrial damage and functional decline in chondrocytes with osteoarthritis, and it serves as a critical link between the aforementioned impairments in mitochondrial biogenesis, imbalances in fusion and fission, and abnormalities in mitochondrial autophagy (Zou et al., 2025) (Figure 3). Under normal conditions, chondrocytes rely on a relatively stable redox environment to maintain mitochondrial respiration, membrane potential, and matrix metabolic balance. However, in the osteoarthritis microenvironment, stimulation by inflammatory factors, aging, abnormal mechanical loading, and metabolic stress can collectively promote the continuous accumulation of ROS, thereby inducing mitochondrial DNA damage, impaired respiratory chain function, and altered membrane permeability (Sun K. et al., 2024). Excessive ROS not only directly damages mitochondrial structure but also further suppresses the antioxidant defense system, causing a persistent imbalance in redox homeostasis both inside and outside the mitochondria, ultimately forming a vicious cycle in which oxidative stress and mitochondrial dysfunction mutually amplify each other (Ge et al., 2022). Reviews have indicated that, among mitochondrial-related pathological changes, oxidative stress is not merely a downstream concomitant phenomenon, but rather a key central mechanism driving the amplification of inflammation, senescence, apoptosis, and matrix degradation in chondrocytes (Fernández-Moreno et al., 2022).

From a mechanistic perspective, excessive accumulation of ROS can exacerbate chondrocyte damage through multiple pathways. On the one hand, damage to the mitochondrial respiratory chain leads to increased electron leakage, which further elevates levels of superoxide anions and peroxides, resulting in lipid peroxidation, protein oxidation, and a decrease in mitochondrial membrane potential (Hou et al., 2026). On the other hand, oxidative stress promotes the activation of inflammation-related signaling pathways such as NF-κB and MAPK, enhancing the expression of IL-1β, TNF-α, and catabolic enzymes, and leading to sustained upregulation of cartilage matrix degradation factors such as MMP13 and ADAMTS5 (Zhang et al., 2024). Furthermore, oxidative stress is closely associated with chondrocyte senescence and ferroptosis. Recent studies indicate that NOX1-mediated oxidative stress promotes chondrocyte ferroptosis and exacerbates the progression of osteoarthritis by inhibiting the Nrf2/HO-1 pathway, suggesting a direct link between insufficient antioxidant defense and mitochondrial damage and the activation of cell death programs (Tao et al., 2024). In human-derived chondrocytes, aging and osteoarthritis are also accompanied by impaired Nrf2 homeostasis and decreased expression of downstream antioxidant proteins, indicating that osteoarthritic chondrocytes are inherently in a vulnerable state that is more susceptible to oxidative damage (Taylor et al., 2023). Therefore, alleviating oxidative stress, restoring antioxidant defenses, and maintaining mitochondrial homeostasis have become key directions in osteoarthritis intervention research (Yang et al., 2025) (Figure 3).

Against this backdrop, TCM-derived monomers have garnered significant attention due to their multi-target regulatory properties. Existing studies indicate that these monomers do not act solely by scavenging free radicals; rather, they more commonly influence stress- and metabolism-related pathways such as Nrf2/HO-1, SIRT1, AMPK, PI3K/Akt, and MAPK, thereby improving mitochondrial function, reducing ROS levels, and alleviating chondrocyte damage (Barbeau-Grégoire et al., 2022). Curcumin is a representative compound that has been extensively studied in this context. Previous research has shown that curcumin can alleviate oxidative stress and mitochondrial dysfunction in osteoarthritis models, reduce ROS accumulation, and improve chondrocyte damage, with its effects linked to the regulation of the p38 MAPK pathway (Zeng et al., 2022). These findings suggest that curcumin is not merely a general anti-inflammatory agent but plays a distinct role in maintaining mitochondrial homeostasis. In addition to curcumin, berberine has also been reported to mitigate the progression of post-traumatic osteoarthritis and alleviate pain through AMPK-related mechanisms, while simultaneously improving pathological changes associated with oxidative stress and metabolic disorders (Wang et al., 2017). Natural polyphenols such as resveratrol and quercetin are similarly believed to alleviate oxidative stress, protect mitochondrial function, and inhibit inflammatory responses in chondrocytes. Although the specific focuses of different studies are not entirely consistent, the overall direction is largely consistent: namely, mitigating mitochondrial damage and its downstream consequences by correcting redox imbalances (Villagrán-Andrade et al., 2024).

Research over the past 2 years has further suggested that certain single compounds derived from traditional Chinese medicine provide more direct experimental evidence regarding their role in regulating oxidative stress and mitochondrial homeostasis. Baichuanhu saponin VI can alleviate mitochondrial dysfunction and oxidative stress, improve chondrocyte damage, and delay the progression of osteoarthritis; its mechanism of action involves the regulation of SIRT1-related pathways (Li et al., 2024). Furthermore, a review on how TCM-derived monomers regulate the axis of oxidative stress and mitochondrial autophagy indicates that various active monomers can collectively maintain chondrocyte mitochondrial homeostasis by reducing ROS burden, restoring membrane potential, promoting the clearance of damaged mitochondria, and mitigating inflammatory amplification (Wang et al., 2020). Overall, research on oxidative stress provides a robust integrative framework for understanding the chondroprotective effects of TCM-derived monomers. Although the aforementioned aspects of mitochondrial biogenesis, dynamics, and autophagy each have their own focus, they are ultimately closely linked to redox imbalance and mitochondrial dysfunction. The role of TCM-derived monomers at this level is primarily manifested through the synergistic action of multiple signaling pathways to reduce ROS accumulation, enhance endogenous antioxidant defenses, stabilize mitochondrial function, and inhibit inflammatory and catabolic responses (Court et al., 2024) (Figure 3). However, there are still some shortcomings in this area. For example, evaluation criteria for oxidative stress and mitochondrial homeostasis vary across studies, and some research remains limited to the levels of ROS and the expression of a few proteins. Verification of mitochondrial function, the extent of oxidative damage, and the causal relationships among signaling pathways remains insufficient. This suggests that future research needs to be further strengthened in terms of model consistency, depth of mechanism exploration, and the integration of *in vivo* and *in vitro* evidence.

## 6 Summary and outlook

In recent years, there has been a growing body of research on the use of single compounds derived from traditional Chinese medicine for the treatment of osteoarthritis, with studies focusing on mitochondrial quality control gradually gaining attention. Current evidence indicates that chondrocytes in osteoarthritis are characterized by widespread impairments in mitochondrial biogenesis, imbalances in fusion and fission, abnormalities in mitochondrial autophagy, and persistently elevated oxidative stress. These processes are interconnected and mutually influential, collectively contributing to chondrocyte functional decline, matrix metabolic disorders, and the progression of joint degeneration. TCM-derived monomers can improve mitochondrial function by regulating these key processes, thereby alleviating inflammatory responses, oxidative damage, aging, and apoptosis, and consequently protecting chondrocytes and delaying the progression of osteoarthritis to some extent. Overall, a relatively clear mechanistic framework has emerged in this research direction.

However, several limitations remain in the current research. First, substantial differences exist among studies in the selected monomers, experimental models, intervention doses, and evaluation criteria, which limit the comparability of results. Second, although many studies suggest that TCM-derived monomers can improve mitochondrial function, the intrinsic links among different aspects of mitochondrial quality control have not been sufficiently clarified, and the depth of mechanistic validation remains variable. In addition, current evidence is still mainly based on *in vitro* experiments and mouse or rat models, with limited long-term studies and translational evidence that more closely reflect clinical disease progression. Furthermore, issues such as low bioavailability and dispersed targets of some TCM-derived monomers may also restrict their further application.

Future research can be advanced in several directions. First, systematic studies of the overall mitochondrial quality control network should be strengthened, focusing not only on changes in individual pathways but also on the coordinated relationships among mitochondrial biogenesis, mitochondrial dynamics, mitophagy, and oxidative stress. Second, a more unified experimental evaluation system should be established, as current studies show considerable heterogeneity in cell models, animal models, intervention protocols, and outcome measures, which limits direct comparison across studies. Third, greater mechanistic depth is needed in future investigations.

Although many studies have reported beneficial effects of TCM-derived monomers on mitochondrial function, causal validation of the relevant mitochondrial quality control pathways remains insufficient in many cases. Fourth, by integrating pharmacokinetics, delivery systems, and local joint intervention strategies, the translational potential of TCM-derived monomers should be further improved. In particular, stronger integration of animal experiments, preclinical studies, and clinically relevant validation will be necessary, since current evidence is still largely based on *in vitro* experiments and rodent models. Overall, the strategy of using TCM-derived monomers to protect chondrocytes by regulating mitochondrial quality control has a solid research foundation and deserves further investigation.
